# The influence of match status on the conditional characteristics of tactical sprint actions in professional soccer players

**DOI:** 10.5114/biolsport.2024.131825

**Published:** 2023-12-13

**Authors:** David Lobo-Triviño, Tomás García-Calvo, Ana Rubio-Morales, Fabio Nevado, Marcos Chena, Juan Angel Piñero-Madrona, Emilio Martín-Ardila, Javier Raya-González

**Affiliations:** 1 Faculty of Sport Sciences, University of Extremadura, Spain; 2 LaLiga Sport Research Section, Madrid, Spain; 3 Spanish Association of Physical Trainers, Spain

**Keywords:** Football, Team-sport, High-intensity running, Tactical analysis, Tracking system

## Abstract

This study aimed to analyse the influence of the match status on the conditional characteristics of tactical sprint actions among Spanish professional soccer players, considering playing positions. Thirty-two Spanish male professional soccer players from a LaLiga Spanish Second Division (LaLiga SmarthBank) team participated in this study. Actions above 85% of the players’ maximum velocity were analysed based on their tactical purpose. These findings provide valuable information regarding the tactical aspects of sprinting in soccer, emphasizing the influence of playing positions and match status on the distribution of tactical sprint actions. No effects of match status were observed for any game phase. However, when tactical actions were individually studied, it was observed that the maximum velocity in Chase actions was higher when the team was winning, while in Press actions, the maximum velocity was higher when the team was losing and in in Run in behind/Penetrate, the maximum velocity was higher in drawing situations compared to losing situations. No effects of match status on the distance covered during sprinting were observed, and regarding duration, significant differences were only observed in Recovery run actions. In addition, the influence of match status is higher when playing positions are considered, although the within playing positions analysis revealed significant differences only in CM players. These findings provide valuable information for the design of specific training drills considering playing positions, suggesting the need to analyse the previous match in order to structure the training load of the microcycle in a comprehensive manner.

## INTRODUCTION

Time-motion analysis has made it possible to understand the physical profiles of professional players during match play [1, 2], helping practitioners to make objective decisions regarding performance and injury risk [3]. Soccer is characterized by the presence of repetitive high intensity actions (HIA) such as jumps, accelerations or sprints interspersed with low-intensity periods [4]. Specifically, Oliva-Lozano et al. [5] revealed that soccer players perform near to 10 sprints per match and cover approximately 19.5 m per sprint. These data confirm that high-intensity sprinting demands have significantly increased in the last decade [6], greatly impacting soccer preparation [7], so further studies on the topic are warranted.

Previous studies have suggested that sprint actions may be considered a prerequisite for successful performance in soccer. This is based on the fact that linear sprints are the most frequent actions in goal situations, creating a shot and evading an opponent [8, 9]. However, the significance of sprint actions extends beyond these specific scenarios, as they are present in critical situations throughout a soccer match, such as goals [7]. Therefore, in order to enhance preparation, it is necessary to perform studies that integrate the conditional with tactical demands of sprint actions. Specifically, Oliva-Lozano et al. [10] and Ju et al. [11] contextualized sprint actions in professional soccer, focusing on tactical roles and determining the associated conditional demands for each action. However, the majority of previous studies have used absolute values to determine sprint actions. Hence, future studies based on individualized velocity thresholds (e.g., 85% of maximum player’s velocity) are required. Moreover, it is important to consider other variables, such as the duration of sprint actions, as highlighted by Oliva-Lozano et al. [10] to optimize the design and prescription of training tasks.

To optimize the aforementioned aims, it is relevant to consider certain contextual variables that influence the sprint demands during matches, with particular emphasis on the match outcome [12–14]. However, prior studies have primarily focused on the final result of the match [10, 11], making it interesting to explore how the match status influences the tactical sprint actions. Another contextual variable with a key role in soccer demands is playing position. In this regard, Oliva-Lozano et al. [10] conducted a study that revealed the significant influence of playing position on the maximum velocity and distance covered during sprinting by Spanish professional soccer players. Specifically, they found that midfielders achieved lower maximum velocities compared to their counterparts. Additionally, these authors reported differences in the frequency and nature of tactical sprint actions performed by players in different playing positions, which has been confirmed by Ju et al. [11] with English professional soccer players. These insights highlight the importance of considering playing position when analysing tactical sprint actions, which allow for a more comprehensive understanding of the demands placed on players in different roles.

Despite the increase in the research focusing on the contextualized analysis of sprint actions in professional soccer, to our knowledge, no studies considering individualized velocity thresholds to analyse tactical sprint demands have been performed. Thus, this study aimed to analyse the influence of the match status on the conditional characteristics of tactical sprint actions among Spanish professional soccer players, considering playing positions. Based on previous studies [10, 11], we hypothesized that match status substantially influences the conditional variables of the tactical sprint actions, being different according to playing positions.

## MATERIALS AND METHODS

### Study design

A retrospective, descriptive longitudinal design was applied to analyse the influence of the match status on the characteristics of sprint actions among Spanish professional soccer players, considering playing positions and conditional and tactical variables. An observation instrument based on the integrated approach of Ju et al. [15] was designed to assess all the sprint actions performed by players. The sprint actions were classified as defence, attack, defence-attack transition (DAT) and attack-defence transition (ADT). Specifically, these actions were categorized according to the classification presented in Table 1.

**TABLE 1:** Descriptions of the variables within the integrated approach.

Actions	Description
***Defense***	
*Chase*	Player chases an opponent who has the ball.
*Press*	Player runs directly towards opposition player on/or receiving the ball, or towards space or players not on/receiving the ball (typically blocking passing channels).
*Recovery run*	Player runs back towards their own goal to be goal side when out of position.
*Close Down/Interception*	Player cuts out pass.
*Collective run*	Most of the players on the team move in the same direction simultaneously

***Attack***	
*Run with ball*	Player moves with the ball either dribbling with small touches or running at speed with fewer ball touches.
*Run in behind/Penetrate*	Player attacks space behind, overtakes and/or unbalances the opposition defense (typically ball is behind).
*Break into the box*	Player enters the opposition’s penalty box in an attempt to receive the ball (typically from a cross – ball in front and wide).
*Over/Underlap*	Player runs from behind to in front of the player on the ball or receiving the ball.
*Move to receive/Exploit the space*	Player moves to receive a pass from a teammate or to create/exploit space (typically comes short or moves wide to receive ball).

Additionally, playing position and conditional variables (i.e., maximum velocity achieved, duration and distance covered in each sprint action) were considered. Specifically, the players were divided into five positions on the field: central defender (CD), wide defender (WD), central midfielder (CM), wide midfielder (WM) and forward (F). To classify the sprint actions based on match status the score at the time of the action was registered as follows: “losing” (L), “drawing” (D) or “winning” (W).

### Participants

Thirty-two professional male soccer players (*M*_age_: 27.2 ± 5.6 years, *M*_height_: 182.3 ± 4.2 cm, *M*_weight_: 78.5 ± 5.5 kg), who competed in LaLiga Spanish Second Division (LaLiga SmarthBank) during the 20/21 season, voluntarily participated in this study. Players belonged to the same squad, which was composed of 6 CD, 3 WD, 11 CM, 8 WM and 4 F. Goalkeepers were excluded due to their specific match demands. A total of 1848 sprint actions in which the players achieved a maximum velocity higher than 85% of their reported maximum velocity during the season were selected [10]. LaLiga provided the data used in the study after participants signed their informed consent. According to the Declaration of Helsinki, the players’ and team confidentiality was ensured. The study received full approval by the Ethics Committee of the University of Extremadura; Vice-Rectorate of Research, Transfer and Innovation – Delegation of the Bioethics and Biosafety Commission (Protocol number: 239/2019).

### Procedures

All the selected sprint actions were independently analysed by four observers, who were members of professional soccer staffs and had experience in soccer research and video analysis. The observers underwent training on the interpretation of these types of actions, and they demonstrated high inter-rater reliability (kappa statistic, κ = 0.85) and an almost perfect intra-rater reliability (kappa statistic, κ = 0.90). The observers categorized the actions according to the possibilities presented in Table 1, and they reached a final agreement on 97% of the actions. For each sprint action, the following variables were provided: match status, playing position, tactical action, maximum velocity, duration and distance covered.

The optical tracking system ChyronHego (TRACAB, New York, US) was used to collect the match data. This multi-camera tracking system consists of 8 super 4K-High Dynamic Range cameras based on a positioning system (Tracab—ChyronHego VTS) which records and analyses X and Y positions for each player from several angles, providing real-time two-dimensional tracking (tracking data are recorded at 25 Hz). Additionally, a customized report was created using Mediacoach software (LaLiga, Madrid, Spain), which synchronized tracking data with the video footage of each match.

### Statistical analysis

All statistical analyses were conducted using RStudio [16]. Considering the characteristics of the sample, organized hierarchically, nested in groups, and with a longitudinal structure, the best procedure to analyse the data is through linear mixed models (LMM). Furthermore, LMM have demonstrated their ability to cope with unbalanced and repeated-measures data [17]. Thus, the cross-classified multilevel models are suitable for data structures that are not purely hierarchical. Consequently, a general multilevel-modelling strategy was applied where fixed and random effects in different steps were included [18]. A two-level hierarchy was modelled for the analysis. The variables related to actions’ characteristics (i.e., maximum velocity, duration and distance) were included as dependent variables in the models. Match status (i.e., losing, drawing and winning) and playing position (i.e., CD, WD, CM, WM and F) were the independent variables included as fixed effects. The player variable was considered as the random effect in the analysis. All LMM were performed using the lm4 package from RStudio [19]. Values were represented as coefficients and standard error (coeff ± SE). SE was selected instead of standard deviation (SD) to show the dispersion of the sample mean if we continued to take values. Statistical significance was set at *p* < 0.05 and *p* < 0.01.

## RESULTS

Table 2 shows the sprint action characteristics according to game phase and match status. No significant differences were observed between match statuses within each game phase regarding maximum velocity, duration and distance.

**TABLE 2:** Action characteristics according to game phase and match status (Coeff ± SE).

	Defense				Attack				DAT				ADT			
	-1	0	1	*df*	-1	0	1	*df*	-1	0	1	*df*	-1	0	1	*df*
Max vel (km · h^−1^)	29.20 ± 0.30	29.20 ± 0.29	29.10 ± 0.32		29.30 ± 0.31	29.40 ± 0.30	29.60 ± 0.34		29.50 ± 0.33	29.50 ± 0.30	29.50 ± 0.35		29.70 ± 0.30	29.50 ± 0.29	29.70 ± 0.32	
Duration (s)	5.81 ± 0.18	5.89 ± 0.14	5.91 ± 0.27		6.14 ± 0.23	6.21 ± 0.17	5.93 ± 0.33		7.45 ± 0.28	7.29 ± 0.19	7.62 ± 0.36		7.22 ± 0.18	7.12 ± 0.14	6.90 ± 0.26	
Distance (m)	34.40 ± 1.11	34.10 ± 0.92	32.80 ± 1.64		37.20 ± 1.39	36.80 ± 1.08	36.20 ± 1.98		46.90 ± 1.69	44.80 ± 1.18	45.40 ± 2.17		44.20 ± 1.13	43.10 ± 0.93	41.70 ± 1.59	

*Notes.* Coeff = coefficients; SE = Standard Error; Defense = defense phase; Attack = attack phase; TDA = defense-attack transition; TAD = attack-defense transition; df = significant differences; -1 = losing; 0 = drawing; 1 = winning; Max vel = maximum velocity achieved in the actions; Duration = average duration of actions; Distance = meters covered during sprint actions; # = significant differences between losing and drawing; $ = significant differences between losing and winning; ¨ = significant differences between drawing and winning; *p < 0.05, p < 0.01.

Sprint actions’ characteristics related to defensive actions are presented in Table 3. The maximum velocity achieved by the Chase action was significantly higher (p < 0.05) when the team was winning compared to when the team was losing or drawing. Similarly, the maximum velocity achieved by Press actions was significantly higher (p < 0.05) when the team was losing than when the team was drawing. Conversely, the distance covered by Recovery run actions was significantly higher (p < 0.05) when the team was losing compared to when the team was winning. No significant differences were observed in the rest of the defensive actions.

**TABLE 3:** Sprint actions characteristics related to defense actions (Coeff ± SE).

	Chase				Press				Recovery run				Close Down/Interception				Collective run			
	-1	0	1	*df*	-1	0	1	df	-1	0	1	*df*	-1	0	1	*df*	-1	0	1	*df*
Max vel (km · h^−1^)	29.80 ± 0.32	29.60 ± 0.30	30.40 ± 0.41	$*¨*	29.40 ± 0.32	28.90 ± 0.30	29.20 ± 0.35	#*	29.40 ± 0.30	29.30 ± 0.29	29.10 ± 0.33		29.20 ± 0.32	29.60 ± 0.30	29.60 ± 0.37		29.50 ± 0.35	29.20 ± 0.31	29.40 ± 0.42	
Duration (s)	6.66 ± 0.27	6.64 ± 0.21	7.03 ± 0.51		5.43 ± 0.27	5.81 ± 0.21	5.83 ± 0.37		7.60 ± 0.21	7.35 ± 0.16	7.21 ± 0.29		5.50 ± 0.28	5.46 ± 0.20	5.34 ± 0.41		7.61 ± 0.37	7.15 ± 0.24	7.38 ± 0.53	
Distance (m)	41.20 ± 1.61	40.60 ± 1.29	44.50 ± 2.96		31.10 ± 1.60	32.30 ± 1.28	30.20 ± 2.16		47.60 ± 1.27	45.20 ± 1.03	42.60 ± 1.74	$ *	32.00 ± 1.70	32.90 ± 1.25	31.50 ± 2.42		46.50 ± 2.16	42.10 ± 1.44	45.90 ± 3.11	

*Notes.* Coeff = coefficients; SE = Standard Error; df = significant differences; Max vel = maximum velocity achieved in the actions; Duration = average duration of actions; Distance = meters covered during sprint actions; -1 = losing; 0 = drawing; 1 = winning; # = significant differences between losing and drawing; $ = significant differences between losing and winning; ¨ = significant differences between drawing and winning;

* *p* < 0.05, *p* < 0.01.

Table 4 shows the summary statistics for action characteristics according to attack action type and match status. The maximum velocity achieved by the Run in behind/Penetrate action was significantly higher (p < 0.01) when the team was drawing than when the team was losing. No significant differences were observed in the rest of the attacking actions.

**TABLE 4:** Sprint actions characteristics related to offensive actions (Coeff ± SE).

	Run with ball				Run in behind/Penetrate				Break into the box				Over/Underlap				Move to receive			
	-1	0	1	*df*	-1	0	1	*df*	-1	0	1	*df*	-1	0	1	*df*	-1	0	1	*df*
Max vel (km · h^−1^)	29.30 ± 0.35	29.30 ± 0.33	29.60 ± 0.44		29.40 ± 0.33	29.90 ± 0.31	29.50 ± 0.36	#	29.20 ± 0.35	29.10 ± 0.32	29.30 ± 0.41		29.90 ± 0.66	29.10 ± 0.40	29.30 ± 0.83		30.20 ± .36	29.10 ± 0.73	-	
Duration (s)	6.64 ± 0.38	6.75 ± 0.29	6.45 ± 0.58		5.78 ± 0.30	6.42 ± 0.24	6.46 ± 0.40		6.82 ± 0.37	6.58 ± 0.27	6.34 ± 0.52		7.83 ± 1.04	7.51 ± 0.50	7.07 ± 1.34		4.52 ± .30	6.60 ± 1.15	-	
Distance (m)	41.90 ± 2.22	41.80 ± 1.77	40.70 ± 3.38		35.10 ± 1.77	38.70 ± 1.45	38.20 ± 2.38		40.10 ± 2.19	37.40 ± 1.63	37.60 ± 3.04		50.30 ± 6.01	45.40 ± 2.94	45.60 ± 7.76		29.60 ± 3.3	39.60 ± 6.69	-	

*Notes.* Coeff = coefficients; SE = Standard Error; df = significant differences; Max vel = maximum velocity achieved in the actions; Duration = average duration of actions; Distance = meters covered during sprint actions; -1 = losing; 0 = drawing; 1 = winning; # = significant differences between losing and drawing; $ = significant differences between losing and winning; ¨ = significant differences between drawing and winning; *p < 0.05, *p* < 0.01.

Table 5 shows the summary statistics for action characteristics according to playing position and match status. Considering the maximum velocity, when the team was drawing, CM achieved lower values than CD (p < 0.01), and when the team was winning, the maximum velocity achieved by CM was significantly lower (p < 0.01) than CD, WD and WM. Considering the duration, when the team was losing the match, the sprint actions performed by CD were significantly shorter (p < 0.05) than CM actions. When the team was drawing the match, CD performed actions significantly shorter (p < 0.01) than WD, CD and WM. Regarding the distance covered, when the team was losing, CD covered significantly less distance (p < 0.05) than the WD, CM and WM. Similarly, when the team was drawing, CD covered significantly less distance during the sprint actions (p < 0.05) than the WD, CM and WM. Finally, when the team was winning, WM covered a significantly greater distance (p < 0.05) than CD, CM and F. Within playing position analysis revealed that maximum velocity achieved in the sprint actions by CM was significantly higher when they were losing than when they were drawing (p < 0.05) or winning (p < 0.01). The duration of the actions performed by CM was significantly longer (p < 0.05) when they were drawing than when they were winning. In relation to the distance covered during the actions, CM covered a significantly greater distance during the sprint actions (p < 0.01) when they were losing than when they were winning the match. No significant differences were observed in the rest of the playing positions.

**TABLE 5:** Sprint actions characteristics according to playing position and match status (Coeff ± SE).

	CD				WD				CM			
	-1	0	1	*df*	-1	0	1	*df*	-1	0	1	*df*
Max vel (km · h^−1^)	29.50 ± 0.35	29.70 ± 0.35	29.90 ± 0.37		29.60 ± 0.40	29.50 ± 0.38	29.90 ± 0.44		29.40 ± 0.32	29.10 ± 0.32	28.70 ± 0.36	#*$¨*
Duration (s)	6.23 ± 0.25	5.91 ± 0.20	6.28 ± 0.34		6.55 ± 0.26	6.75 ± 0.19	6.56 ± 0.42		6.91 ± 0.22	7.12 ± 0.17	6.25 ± 0.32	¨*
Distance (m)	36.50 ± 1.67	35.20 ± 1.42	37.10 ± 2.16		41.60 ± 1.86	42.50 ± 1.53	41.40 ± 2.70		41.30 ± 1.44	39.80 ± 1.25	35.30 ± 2.04	$¨^*^
	**WM**				**F**				***df***			
	-1	0	1	*df*	-1	0	1	*df*	-1	0	1	
Max vel (km · h^−1^)	29.60 ± 0.34	29.50 ± 0.33	29.70 ± 0.37		29.30 ± 0.38	29.30 ± 0.36	29.30 ± 0.34			b	beh	
Duration (s)	6.60 ± 0.23	6.74 ± 0.18	6.84 ± 0.34		6.34 ± 0.27	6.18 ± 0.19	6.76 ± 0.35		b*	abcg^*^i		
Distance (m)	41.10 ± 1.55	41.50 ± 1.34	43.30 ± 2.16		38.60 ± 1.83	37.01 ± 1.43	36.60 ± 2.24		a^*^b^*^c^*^	ab^*^cg^*^j^*^	c^*^hj^*^	

*Notes.* Coeff = coefficients; SE = Standard Error; CD = central defender; WD = wide defender; CM = central midfielder; WM = wide midfielder; F = Forward; df = significant differences; -1 = losing; 0 = drawing; 1 = winning; Max vel = maximum velocity achieved in the actions; Duration = average duration of actions; Distance = meters covered during sprint actions; # = significant differences between losing and drawing; $ = significant differences between losing and winning; ¨ = significant differences between drawing and winning; a = significant differences between CD and WD; b = significant differences between CD and CM; c = significant differences between CD and WM; d = significant differences between CD and F; e = significant differences between WD and CM; f = significant differences between WD and WM; g = significant differences between WD and F; h = significant differences between CM and WM; i = significant differences between CM and F; j = significant differences between WM and F;

* *p* < 0.05, *p* < 0.01.

## DISCUSSION

This study aimed to analyse the influence of the match status on the conditional characteristics of tactical sprint actions among Spanish professional soccer players, considering playing positions. This is the first study to investigate the effects of match status on tactical sprint actions in relation to conditional variables. The results obtained show that the conditional characteristics of tactical sprint actions are lightly influenced by match status, although when playing positions are considered this influence is increased.

Soccer is divided into four game phases (i.e., defence, attack, DAT and ADT) that generally have different conditional demands [20]. However, these demands related to sprint actions remain constant regardless of the match status, so an analysis based on specific tactical actions seems necessary. Previous studies have analysed the differences in conditional variables of tactical sprint actions [10, 11], mainly considering playing positions, but none of them have done so taking into account the match status. Regarding defence action, the maximum velocity achieved during Chase actions was higher when the team was winning compared to when the team was losing or drawing. This could be attributed to a greater emphasis on defending a positive result, which would generate a faster Chase action. Additionally, when the team was losing, Press actions required higher maximum velocity than when the team was drawing, likely due to the increased height and intensity in pressure to regain possession quickly and increase the chances of drawing the game. Related to this, a longer duration in Recovery run sprint action was observed when the team was losing compared to when the team was winning. The increase in pressure when the team is losing leads to Recovery run actions starting from positions further away from their own goal, resulting in longer durations. Regarding attack actions, only differences were observed in Run in behind/Penetrate, with a higher maximum velocity when the team was drawing than when the team was losing. These findings suggest the need to structure the training load of the microcycle according to the development of the previous match, which must be comprehensively analysed.

Some authors have observed that playing position has a significant effect on the maximum velocity and distance covered during sprinting [10]. However, these authors selected sprint actions based on an absolute value (i.e., > 30 km · h^−1^), and did not consider the match status, making comparisons difficult. In our study, it was observed that CD employed shorter durations to cover sprint actions than CM when the team was losing. Similarly, the distance covered by CD in these conditions was lower than WD, CM and WM. These differences could be supported by the fact that when the team was losing, the majority of playing positions focused on attacking, resulting in higher sprint efforts in terms of duration and distance for those playing positions. When the team was drawing, CD achieved higher maximum velocities than CM, while the duration and the distance covered during sprint actions by CD is shorter compared to WD, CM, WM. In this line, lower distances and durations were observed for F compared to WD and CM. Similarly to when the team was losing, higher sprint efforts are imposed on WD, WM and CM, although CD must increase the maximum velocity of this sprint actions to cover gaps left by teammates while attacking. Finally, when the team was winning, lower maximum velocities were observed in CM sprint actions than CD, WD and WM, possibly because these players focus on maintaining their position on the field, avoiding unnecessary risks when they are winning. Likewise, the distance covered by WM was shorter than F, CD and CM, as the sprint actions made by these players may be reduced in an effort to maintain their position on the field. Within playing positions differences were only observed in CM. In this regard, the maximum velocity is higher when drawing or winning compared to when losing, and also higher when drawing compared to when winning. Additionally, the distance and the duration of sprint actions are reduced when the team is winning. This could be due to the specific role of CM, which involves a combination of defensive and attacking actions that are influenced by the match status [21].

This study has some limitations that should be acknowledged. The main one is that only male soccer players were analysed. Thus, further studies involving female players are required. Likewise, a single team was studied; since each team has its specific characteristics, these findings should be considered with caution. However, a recent study compared match demands between First and Second Division teams [22], obtaining similar results, which could indicate the possibility of generalizing our results. Finally, match-related contextual variables such as team formation, team level, style of play or players’ age were not considered.

## CONCLUSIONS

This study provides a novel approach to achieving a comprehensive analysis of tactical sprint actions in professional soccer by incorporating the effects of match status. In this regard, no effects of match status were observed for any game phase. However, when tactical actions were individually studied, it was observed that the maximum velocity in Chase actions was higher when the team was winning, while in Press actions, the maximum velocity was higher when the team was losing and in Run in behind/Penetrate, the maximum velocity was higher in drawing situations compared to losing situations. No effects of match status on the distance covered during sprinting were observed, and regarding duration, significant differences were only observed in Recovery run actions. However, the influence of match status is greater when playing positions are considered, revealing significant differences in the within playing positions analysis only in CM players. These findings provide valuable information regarding how match status influences sprint actions in professional soccer players. In a practical approach, strength and conditioning coaches must design specific training drills considering playing positions and based on the analysis of the previous match in order to structure the training load of the microcycle in a comprehensive manner, transitional activities being an interesting option [23].
